# The use of non-steroidal anti-inflammatory drugs (NSAIDs) in COVID-19

**DOI:** 10.1038/s41533-022-00300-z

**Published:** 2022-09-21

**Authors:** Pamela Kushner, Bill H. McCarberg, Laurent Grange, Anton Kolosov, Anela Lihic Haveric, Vincent Zucal, Richard Petruschke, Stephane Bissonnette

**Affiliations:** 1 Kushner Wellness Center, Los Angeles, CA USA; 2 Department of Family Medicine, University of California, Irvine, CA USA; 3 Department of Family Medicine, University of California at San Diego School of Medicine, San Diego, CA USA; 4 Rheumatology Department, Grenoble-Alpes University Hospital, Echirolles, France; 5 President of the French League Against Rheumatism (AFLAR), Paris, France; 6 Medical Affairs, GSK Consumer Healthcare, Rochester Park, Singapore, Singapore; 7 Consumer Safety, GSK Consumer Healthcare, Nyon, Switzerland; 8 Consumer Safety, GSK Consumer Healthcare, Warren, NJ USA; 9 Medical Affairs, GSK Consumer Healthcare, Warren, NJ USA

**Keywords:** Respiratory tract diseases, Therapeutics

## Abstract

Early in the COVID-19 pandemic, anecdotal reports emerged suggesting non-steroidal anti-inflammatory drugs (NSAIDs) may increase susceptibility to infection and adversely impact clinical outcomes. This narrative literature review (March 2020–July 2021) attempted to clarify the relationship between NSAID use and COVID-19 outcomes related to disease susceptibility or severity. Twenty-four relevant publications (covering 25 studies) reporting original research data were identified; all were observational cohort studies, and eight were described as retrospective. Overall, these studies are consistent in showing that NSAIDs neither increase the likelihood of SARS-CoV-2 infection nor worsen outcomes in patients with COVID-19. This is reflected in current recommendations from major public health authorities across the world, which support NSAID use for analgesic or antipyretic treatment during COVID-19. Thus, there is no basis on which to restrict or prohibit use of these drugs by consumers or patients to manage their health conditions and symptoms during the pandemic.

## Introduction

Severe acute respiratory syndrome coronavirus 2 (SARS-CoV-2) was identified as the cause of coronavirus disease 2019 (COVID-19) in January 2020^1^. Soon afterwards (on March 11, 2020), the World Health Organization (WHO) declared the disease a pandemic^2^. It has been estimated that around 35% of COVID-19 infections are asymptomatic^3^. In symptomatic cases, clinical manifestations include fever, cough, body aches and pains, headache, sore throat, gastrointestinal symptoms, acute respiratory disease, pneumonia, and loss of smell or taste^4–6^. Symptoms range in severity from mild to extremely severe, and patients with severe symptoms may require hospitalization and ventilation. In July 2021, the total number of global deaths related to COVID-19 had almost reached 4 million^7^. Global data on hospitalization rates are lacking but, in the USA between February 2020 and March 2021, there were an estimated 97.1 million symptomatic illnesses and 5.6 million hospitalizations related to COVID-19^8^. In the early stages of the pandemic, best practice for managing hospitalized patients was undefined. Since then, treatment guidelines have been developed that include recommendations on the use of medications known to be beneficial^9–12^.

Non-steroidal anti-inflammatory drugs (NSAIDs) were among the medications used in the early stages/phase of the pandemic to manage COVID-19 in both the inpatient and outpatient settings. NSAIDs have diverse structural and pharmacodynamic profiles, but similar modes of action. Their main pharmacological action is inhibition of cyclooxygenase (COX), also known as prostaglandin endoperoxide H synthase^13^. These drugs are commonly used for their analgesic, anti-inflammatory, or antipyretic effect, and were, therefore, considered as primary candidates for treating a number of the symptoms of COVID-19 (i.e., fever, body aches, headache). Despite this, there were early, unconfirmed, anecdotal reports that NSAIDs could increase susceptibility to infection or aggravate the disease^14–16^. On March 14, 2020, the French Health Minister addressed this topic in a tweet^17^. Based on limited preclinical data, it was hypothesized that NSAIDs (particularly ibuprofen, which is available worldwide over the counter [OTC] and the most commonly used NSAID) may be detrimental to patients with COVID-19 due to upregulation of angiotensin-converting enzyme (ACE) 2 expression, which, in turn, may facilitate viral entry into host cells and enhance pathogenicity of the viral load^14,18^. This concern was reported in the British Medical Journal, and despite a lack of evidence to confirm such risk, there was a significant reduction in NSAID use at a time when they could have benefitted those infected with COVID-19^19–21^. In England, the National Health Service suggested that, in the absence of clear evidence, patients should be advised to take paracetamol to treat the symptoms of COVID-19 in preference to NSAIDs^22^. Around the same time, the lack of scientific data to support an increased risk of SARS-CoV-2 infection or COVID-19 severity with ibuprofen was acknowledged by other authorities, including the European Medicines Agency (EMA)^15,22,23^.

The potential for NSAIDs to adversely affect the occurrence or severity of COVID-19 via upregulated expression of ACE-2 has been disputed, with several studies reporting that previous and concurrent use of ACE inhibitors or angiotensin receptor blockers, which are widely known to cause upregulated expression of ACE-2, has no impact on COVID-19 frequency or severity^24–27^. Recent experimental data suggest that NSAIDs do not affect ACE-2 expression or viral entry into host cells in humans and mice^28^. Instead, the key effects of NSAIDs appear attributable to the modulation of the inflammatory and humoral immune responses to SARS-CoV-2, as shown in mice studies where NSAIDs limit the production of proinflammatory cytokines and may reduce levels of neutralizing antibodies^28^. Investigation of this effect in humans, and whether the timing of NSAID use affects COVID-19 pathogenesis, is an important area for further research^28^.

To clarify the relationship between NSAID use and outcomes relating to COVID-19, we performed a comprehensive review of the published literature evaluating associations between NSAID use and COVID-19 outcomes related to disease susceptibility or severity.

## Literature searches

Literature searches were carried out to identify clinical studies, case reports, or review articles relating to the potential effects of NSAIDs on clinical outcomes in human patients with COVID-19 or similar SARS-related coronaviruses. The literature search covered the period from March 2020 to July 2021. The following search string was used to search Embase and Medline:

(“COVID 19”/exp OR “COVID-19” OR “SARS-related coronavirus”/exp OR “SARS-related coronavirus” OR “severe acute respiratory syndrome”/exp OR “severe acute respiratory syndrome”) AND (“ibuprofen”/exp OR ibuprofen or NSAID)) AND (“case report”/de OR “clinical article”/de OR “clinical trial”/de OR “human”/de).

The articles retrieved from the two databases were independently reviewed for relevance by three reviewers. All primary research articles presenting clinical or pharmacoepidemiologic data on NSAID use in the context of COVID-19 were included. Data on outcomes, study population, type of NSAID and dose regimen, and statistical methodology were extracted. Relevant review articles were also checked for additional studies not identified by the literature search.

## Results

In total, 24 relevant publications (covering 25 studies) reporting original research data were identified (Table 1). All of these were observational cohort studies, and eight were described as retrospective. No randomized controlled trials were identified. Nineteen of the studies were conducted in patients with COVID-19 or SARS-CoV-2 infection, and the number of participants ranged from 103 to 2.74 million. In addition, a meta-analysis that included 2414 patients from three studies was retrieved^29^.

**Table 1:** Summary of included studies.

Reference	Country/region	Number of participants	Setting/participant type	Study type	Study objective	Key findings
*NSAIDS—general*						
Blanch-Rubió et al. (2020)^30^	Spain	2102	Patients with non-inflammatory rheumatic conditions attending an outpatient service	Cross-sectional	To investigate the influence of treatments for osteoporosis, OA, and fibromyalgia on COVID-19 incidence and clinical expression	No association between COVID-19 and chronic NSAID use identified. Values for aRR of COVID-19 among chronic NSAID users vs. controls, calculated using two different statistical models, were 0.94 (95% CI 0.57–1.56) and 0.95 (95% CI 0.58–1.55)
Drew et al. (2021)^31^ Pre-print	US, UK, Sweden	2.74 million	General public	Prospective survey	To investigate whether NSAIDs are associated with incident COVID-19 outcomes	No evidence to support increased risk for COVID-19 in those that regularly use aspirin or non-aspirin NSAIDs. After adjustment for lifestyle factors, comorbidities, and baseline symptoms, any NSAID use was not associated with risk for testing COVID-19 positive (HR 1.02 [95% CI 0.94, 1.10]). Results were similar for those seeking healthcare for COVID-19 and were not substantially different according to lifestyle and sociodemographic factors
Chandan et al. (2021)^32^	UK	25,659	Primary care patients with OA	Propensity score-matched cohort study with active comparators	To identify whether active use of NSAIDs increases susceptibility to developing COVID-19 compared to other common analgesics	No increase in the risk of suspected or confirmed COVID-19 or mortality was observed among patients with OA in a primary care setting who were prescribed NSAIDs as compared to those who received comparator drugs Adjusted HRs for suspected or confirmed COVID-19 among the unmatched and propensity score-matched OA cohorts, using data from clinical consultations in primary care settings, were 0.82 (95% CI 0.62–1.10) and 0.79 (95% CI 0.57–1.11), respectively. Adjusted HRs for the risk of all-cause mortality were 0.97 (95% CI 0.75–1.27) and 0.85 (95% CI 0.61–1.20), respectively. There was no effect modification by age or sex
Wong et al. (2021)^33^	England	Study 1: 2.5 million Study 2: 1.7 million	Study 1: General population with at least one NSAID prescription in the last 3 years Study 2: individuals with RA/OA	Cohort studies	To assess the association between routinely prescribed NSAIDs and deaths from COVID-19	No evidence of a harmful effect of routinely prescribed NSAIDs on COVID-19 related deaths Study 1: no evidence of difference in risk of COVID-19 related death associated with current NSAID use (HR 0.96 [95% CI 0.80–1.14]) Study 2: a lower risk of COVID-19 related death (HR 0.78 [95% CI 0.64–0.94]) associated with current use of NSAID vs. non-use
Hwang et al. (2020)^34^	South Korea	103	Adult in-patients diagnosed with COVID-19	Retrospective cohort study	To identify differences in demographic data between COVID-19 survivors and non-survivors	NSAID use not significantly associated with death of COVID-19 patients. Numbers of patients with a history of taking NSAIDs were 3/77 (4%) among survivors and 2/26 (8%) among non-survivors (*p* = 0.436)
Hasseli et al. (2021)^35^	Germany	468	Patients with inflammatory rheumatic diseases infected with SARS-CoV-2	National registry	To describe patients with rheumatic and musculoskeletal diseases according to their COVID-19 severity and to identify risk factors for hospitalization	Univariate analyses indicated that NSAID use did not significantly affect the risk of COVID-19 related hospitalization
Gianfrancesco et al. (2020)^36^	Europe	600	Individuals with rheumatic disease and COVID-19	International registry	To describe the demographic and clinical characteristics associated with COVID-19 hospitalization status in people with rheumatic disease	NSAIDs use for pre-existing chronic conditions is not associated with increased risk of severe outcome in COVID-19 infection. NSAID use was reported less frequently among hospitalized patients than non-hospitalized patients (16% vs. 25%, *p* = 0.02; aOR 0.64 [95% CI 0.39–1.06])
Jehi et al. (2020)^37^	US	4536	Single healthcare system in Ohio and Florida COVID-19-positive patients	Retrospective cohort study	To characterize patients hospitalized with COVID-19 and their outcomes, and develop a statistical model for prediction of hospitalization risk in newly diagnosed patients	4536 patients tested positive for SARS-CoV-2 during the study period. Of those, 958 (21.1%) required hospitalization Hospitalization risk increased with use of certain medications, including NSAIDs
Abu Esba et al. (2021)^38^	Saudi Arabia	503	Adult patients diagnosed with COVID-19	Prospective cohort study	To assess the association of acute and chronic use of NSAIDs with worse COVID-19 outcomes	Acute or chronic NSAID use was not associated with a greater risk of mortality (aHR 0.492 [95% CI 0.178–1.362; *p* = 0.1721]) NSAID users did not have a significantly longer time to clinical improvement or length of stay
Lund et al. (2020)^39^	Denmark	9236	Individuals who tested positive for SARS-CoV-2	Population-based cohort study	To study whether use of NSAIDs is associated with adverse outcomes and mortality during SARS-CoV-2 infection	Primary outcome: NSAID treatment was not associated with 30-day mortality in the crude unmatched analyses or adjusted analyses; for the adjusted analyses, the 30-day mortality RR was 1.02 (95% CI 0.57–1.82) Secondary outcomes: after adjustment, NSAID use was not associated with hospitalization (RR 1.16 [95% CI 0.87–1.53]), ICU admission (RR 1.04 [95% CI 0.54–2.02]), mechanical ventilation (RR 1.14 [95% CI 0.56–2.30]), or renal replacement therapy (RR 0.86 [95% CI 0.24–3.09])
Park et al. (2021)^41^	South Korea	2763 (794 participants included in propensity score-matched analysis)	Patients diagnosed with COVID-19	Retrospective review of insurance benefit claims	To investigate the association between NSAID use before diagnosis of COVID-19 and mortality afterwards	Use of NSAIDs before COVID-19 diagnosis was not associated with increased mortality or need for ventilator care In the propensity score-matched analysis, all-cause mortality in the NSAIDs group was not significantly different from that in the paracetamol group (4.0% vs. 3.0%; HR 1.33 [95% CI 0.63–2.88]). The rate of ventilator care also showed no significant difference between the two groups (2.0% vs. 1.3%; HR 1.60 [95% CI 0.53–5.30])
Reese et al. (2021)^42^ Pre-print	US	250,533	Individuals testing positive for COVID-19	Retrospective observational study of electronic health record data	To assess associations of eight COX inhibitors with clinical severity and mortality of COVID-19 patients	This study found an association with increased COVID-19 severity for five COX inhibitors (aspirin, ibuprofen, ketorolac, naproxen, and paracetamol). The OR relating to aspirin use in the OA cohort (*n* = 2266 patients) was 3.25 (95% CI 2.76–3.83). Aspirin and paracetamol were also associated with increased mortality. There was no evidence of an association with increased COVID-19 severity or mortality for diclofenac, meloxicam, or celecoxib
Imam et al. (2020)^40^	US	1305	Inpatients diagnosed with COVID-19	Retrospective review of hospital records	To study patient demographics and their impact on in-hospital mortality in a large cohort of COVID-19 patients	Patients using NSAIDs prior to hospitalization had lower odds of mortality (OR 0.55 [95% CI 0.39–0.78], *p* = 0.001), a finding which remained present on multivariate regression analysis (aOR 0.56 [95% CI 0.40–0.82])
Bruce et al. (2020)^43^	UK	1222	Adult patients with COVID-19 admitted to hospital	Prospective observational study	To examine the association between prior use of NSAIDs and mortality and length of hospital stay in patients with COVID-19	No significant negative effect of NSAID use on mortality or time to discharge In-hospital mortality was 25.9% (*n* = 14) in NSAID users and 29.5% (*n* = 344) among non-users (*p* = 0.578). In multivariable analysis, no association between pre-admission NSAID use and time to mortality, aHR = 0.89 (95% CI 0.52–1.53, *p* = 0.67) No association between pre-admission NSAID use and day-7 mortality (aOR = 0.79, *p* = 0.60) or time to discharge (aHR = 0.89, *p* = 0.58)
Drake et al. (2021)^44^	UK	78,674	Hospitalized patients with confirmed or highly suspected SARS-CoV-2 infection leading to COVID-19	Prospective cohort study	To characterize the safety of NSAIDs and identify whether pre-existing NSAID use was associated with increased severity of COVID-19 disease	After adjusting for explanatory variables, NSAID use was not associated with worse in-hospital mortality (matched OR 0.95 [95% CI 0.84–1.07]; *p* = 0.35), critical care admission (1.01 [95% CI 0.87–1.17]; *p* = 0.89), requirement for invasive ventilation (0.96 [95% CI 0.80–1.17]; *p* = 0.69), requirement for non-invasive ventilation (1.12 [95% CI 0.96–1.32]; *p* = 0.14), requirement for oxygen (1.00 [95% CI 0.89–1.12]; *p* = 0.97), or occurrence of acute kidney injury (1.08 [95% CI 0.92–1.26]; *p* = 0.33)
Jeong et al. (2020)^46^	South Korea	1824	Patients hospitalized with COVID-19	Cohort study	To examine the association between NSAIDs use compared to non-use and worsened clinical outcomes	NSAID use (prescription of NSAIDs during the 7 days before hospitalization; *n* = 285) was associated with increased risk of primary outcome (death, ICU admission, mechanical ventilation, or sepsis; aOR 1.54 [95% CI 1.11–2.15]), and secondary outcome (risk of cardiovascular or renal complications; aOR 2.64 [95% CI 1.67–4.16])
*Ibuprofen*						
Wong et al. (2021)^33^	England	Study 1: 2.5 million Study 2: 1.7 million	Study 1: General population with at least one NSAID prescription in the last 3 years Study 2: individuals with RA/OA	Cohort studies	To assess the association between routinely prescribed NSAIDs and deaths from COVID-19	Study 1: aHR for COVID-19 related death among current users of ibuprofen vs. non-NSAID users was 1.23 (95% CI 0.90–1.68) Study 2: aHR for COVID-19 related death among current users of ibuprofen vs. non-NSAID users was 0.83 (95% CI 0.56–1.25)
Rinott et al. (2020)^47^	Israel	403	Individuals with COVID-19	Retrospective cohort study	To evaluate whether ibuprofen use in individuals with COVID-19 is associated with more severe disease, compared with individuals using paracetamol or no antipyretics	In the ibuprofen group, three (3.4%) patients died, whereas in the non-ibuprofen group, nine (2.8%) patients died (*p* = 0.95). Nine (10.3%) patients from the ibuprofen group needed respiratory support, compared with 35 (11%) from the non-ibuprofen group (*p* = 1). When compared with exclusive paracetamol users, no differences were observed in mortality rates or the need for respiratory support among patients using ibuprofen
Kragholm et al. (2020)^48^	Denmark	1872	Individuals with COVID-19	Nationwide registry study	To determine the association between recent ibuprofen exposure in COVID-19 patients and severe infection	No significant (*p* = 0.74) association between ibuprofen prescription claims and severe COVID-19; standardized absolute risks of the composite outcome for ibuprofen-prescribed vs. non-ibuprofen patients were 16.3% (95% CI 12.1–20.6) vs. 17.0% (95% CI 16.0–18.1)
Abu Esba et al. (2021)^38^	Saudi Arabia	503	Adult patients diagnosed with COVID-19	Prospective cohort study	To assess the association of acute and chronic use of NSAIDs with worse COVID-19 outcomes	Acute use of ibuprofen was not associated with a greater risk of mortality relative to non-use of NSAIDs (aHR 0.632 [95% CI 0.073–5.441; *p* = 0.6758]) Acute ibuprofen use was not associated with a higher risk of hospital admission compared to non-NSAID users
Castro et al. (2020)^49^ Pre-print	US	12,818	Patients with COVID-19	Cohort study using electronic health records	To identify commonly prescribed medications that may be associated with lesser risk of morbidity with COVID-19	Ibuprofen was significantly associated with diminished risk for hospitalization (OR 0.73 [95% CI 0.64–0.84]). Among hospitalized patients, ibuprofen was significantly associated with decreased odds of requiring ICU (OR 0.70 [95% CI 0.56–0.86]) and mortality (0.73 [95% CI 0.56–0.96])
Choi et al. (2020)^50^	South Korea	293	Individuals hospitalized with early-stage COVID-19	Cohort study	To evaluate risk factors for disease severity in early stages of COVID-19	Before propensity-score matching, there was an increased likelihood (*p* = 0.003) of ibuprofen use among individuals with COVID-19 disease progression compared with those exhibiting improvement or stabilization. After propensity-score matching, ibuprofen use was not a risk factor for progression
Samimagham et al. (2020)^51^	Iran	158	Hospitalized adult patients with COVID-19	Cross-sectional	To investigate the effect of ibuprofen on the severity of COVID-19 and mortality caused by the disease	A significant (*p* < 0.001) relationship was identified between history of ibuprofen consumption before COVID-19 and the severity of the disease, as well as the mortality rate of patients with COVID-19
*Aspirin*						
Drew et al. (2021)^31^ Pre-print	US, UK, Sweden	2.74 million	General public	Prospective survey	To investigate whether NSAIDs are associated with incident COVID-19 outcomes	No evidence to support increased risk for COVID-19 in those that regularly use aspirin. The risk of contracting COVID-19 in this group was not significantly different from that among individuals not taking any NSAIDs, either before or after adjustment for confounders (HR after adjustment: 1.03 [95% CI 0.83–1.28])
Chow et al. (2021)^52^	US	412	Hospitalized adult patients with COVID-19	Retrospective, observational cohort study	To evaluate if aspirin use is associated with the need for mechanical ventilation in adult patients hospitalized with COVID-19	Aspirin use was independently associated with a lower risk of mechanical ventilation, ICU admission, and in-hospital mortality After adjustment for confounding variables, the risk of ICU admission was reduced by 43% (HR 0.57 [95% CI 0.38–0.85]) in users of aspirin compared with non-users. Similar reductions in the risks of mechanical ventilation (HR 0.56 [95% CI 0.37–0.85]) and in-hospital mortality (HR 0.53 [95% CI 0.31–0.90]) were also observed

*aHR* adjusted hazard ratio*, aOR* adjusted odds ratio*, aRR* adjusted relative risk, *CI* confidence interval, *COVID-19* coronavirus disease 19, *COX* cyclooxygenase, *HR* hazard ratio, *ICU* intensive care unit, *NSAIDs* non-steroidal anti-inflammatory drugs, *OA* osteoarthritis, *OR* odds ratio, *RA* rheumatoid arthritis, *RR* risk ratio*, SARS-CoV-2* severe acute respiratory syndrome coronavirus 2.

### NSAIDs—general

A total of 17 publications (covering 18 studies) reported data on the effects of treatment with one or multiple NSAIDs, either prescribed or self-obtained OTC, on outcomes relating to COVID-19. Three of these compared the risks of contracting COVID-19 among individuals treated with NSAIDs with those not receiving NSAIDs. In a cross-sectional study of patients (*n* = 2102, *n* = 318 classified as chronic NSAID users) treated at the outpatient Rheumatology Service of Hospital del Mar, Barcelona, Spain, there was no association between the COVID-19 infection rate and chronic NSAID use^30^. Drew et al. analyzed data from 2.74 million users of the COVID Symptom Study smartphone application from the US, UK, and Sweden^31^. Initial analysis included 8966 COVID-19-positive test results documented over >60 million person-days of follow-up, and NSAID use was associated with a modest increase in the risk of testing positive for COVID-19 (hazard ratio [HR] 1.22 [95% confidence interval (CI) 1.13–1.32]). However, after adjusting for potential confounders (lifestyle factors, comorbidities, and baseline symptoms), NSAID use was not associated with any change in this risk (HR 1.02 [95% CI 0.94–1.10]). Chandan et al. used a large UK primary care data set (*n* ≥ 25,000 adults with osteoarthritis) to compare susceptibility to developing suspected or confirmed COVID-19 among patients prescribed NSAIDs vs. other common analgesics (either paracetamol/codeine or paracetamol/dihydrocodeine)^32^. They found no increase in the risk of suspected/confirmed COVID-19, and no increase in all-cause mortality, among those who were prescribed NSAIDs compared with those who received the comparator drugs.

Two large English cohort studies assessing NSAID use and deaths from COVID-19 (in the general population and patients with rheumatoid arthritis or osteoarthritis) were reported in a single publication^33^. In the general population (2.5 million participants), prescribed NSAID use did not affect the risk of COVID-related death. Among 1.7 million patients with rheumatoid arthritis or osteoarthritis, analysis with adjustment for confounders showed a lower risk of death from COVID-19 among users of prescribed NSAIDs (HR 0.78 [95% CI 0.64–0.94]). A small cohort study from South Korea (*n* = 103 adult patients with COVID-19) evaluated factors that increase the COVID-19 patient death rate and showed no statistically significant difference in NSAID use between the survivor and non-survivor COVID-19 patients^34^.

Three studies investigated the relationship between hospitalization for COVID-19 and NSAID use. In an analysis of the German national registry of patients with inflammatory rheumatic and musculoskeletal disease, NSAID treatment was not identified as a risk factor for COVID-19 related hospitalization^35^. NSAID therapy was reported in 19.1% of hospitalized patients and 25.4% of non-hospitalized population; univariate analysis indicated that NSAID use had no significant effect on the risk of COVID-19 related hospitalization. Similarly, a European international study of rheumatic disease patients with COVID-19 showed that NSAID therapy was less common in a hospitalized vs. non-hospitalized population (16% vs. 25%)^36^. A retrospective cohort study of electronic health records for >4500 US patients with COVID-19 did, however, find a statistically significant association between NSAID use and hospitalization^37^.

A prospective cohort study reported by Abu Esba et al. assessed the association between NSAID use and COVID-19 outcomes (including death, hospital admission, severity, time to clinical improvement, oxygen requirement, and length of hospital stay) in Saudi Arabian adult patients^38^. The study concluded that acute or chronic use of NSAIDs was not associated with worse COVID-19 outcomes compared to non-NSAID users. A Danish nationwide cohort study also assessed the association between NSAID use (defined as a filled prescription up to 30 days before the patient had a positive test for SARS-CoV-2) and various COVID-19 outcomes^39^. Treatment with NSAIDs was not associated with 30-day mortality, risk of hospitalization, intensive care unit (ICU) admission, mechanical ventilation, or renal replacement therapy. In addition, a retrospective, multi-center cohort study conducted in the US (*n* = 1305 patients with COVID-19) showed that NSAID use prior to admission was not associated with development of renal failure or increased mortality^40^. Indeed, patients using NSAIDs prior to hospitalization had lower odds of mortality (odds ratio [OR]: 0.55 [95% CI 0.39–0.78]), a finding which remained present on multivariate regression analysis (adjusted OR 0.56 [95% CI 0.40–0.82]). In a retrospective South Korean study, Park et al. conducted propensity score-matched analysis to compare outcomes in COVID-19 patients treated with NSAIDs vs. those receiving paracetamol^41^. No significant differences between the two groups were observed in all-cause mortality or the incidence of ventilator care.

The largest study of COVID-19 patients identified was a US retrospective cohort study using electronic health record data from >250,000 patients^42^. Potential associations between eight COX inhibitors (paracetamol, aspirin, celecoxib, diclofenac, ibuprofen, ketorolac, meloxicam, naproxen) and COVID-19 severity were assessed. The study reported an association with increased COVID-19 severity for five of the eight agents tested (aspirin, ibuprofen, ketorolac, naproxen, and paracetamol); no significant associations were found for diclofenac, meloxicam, and celecoxib, which display selectivity for inhibition of COX-2. Aspirin and paracetamol (the latter of which is not considered to be COX-selective) were also associated with increased mortality.

A multi-center, observational study conducted in the UK examined the association between use of NSAIDs and outcomes in hospitalized patients (*n* = 1222) with COVID-19 admitted to eight UK hospitals^34,43–45^. Univariate analysis suggested a modest benefit of prior NSAID use, while multivariable analyses indicated no association between prior NSAID use and time to mortality or length of hospital stay. Another UK study enrolled 78,674 patients across 255 healthcare facilities to assess whether pre-existing NSAID use (within the 2 weeks before hospital admission) was associated with increased severity of COVID-19 disease^34,43–45^. The primary outcome was in-hospital mortality, and secondary outcomes included disease severity at presentation, admission to critical care, receipt of invasive ventilation, receipt of non-invasive ventilation, use of supplementary oxygen, and acute kidney injury. After adjusting for explanatory variables, NSAID use was not associated with worsening of any COVID-19 disease outcomes. A South Korean study of 1824 patients hospitalized with COVID-19 reported higher risk of two composite adverse outcomes among NSAID users (individuals prescribed NSAIDs during the 7 days before hospitalization)^46^. For the first outcome (in-hospital death, ICU admission, mechanical ventilation use, or sepsis), the adjusted OR was 1.54 (95% CI 1.11–2.15) and, for the second outcome (cardiovascular or renal complications), it was 2.64 (95% CI 1.67–4.16).

A meta-analysis by Kow and Hasan included data from 2414 patients across three studies that compared COVID-19 patients who were users or non-users of NSAIDs^29^. A pooled HR of 0.86 (95% CI 0.49–1.51) demonstrated no significant difference in the risk of fatality between NSAID users and non-NSAID users.

### Ibuprofen

The two large cohort studies by Wong et al. also reported data specifically for ibuprofen^33^; in both, the general population and patients with rheumatoid arthritis or osteoarthritis, risk of death from COVID-19 was not significantly different between users of ibuprofen vs. other NSAIDs.

Several other studies have assessed the impact of ibuprofen use on the risk of severe COVID-19. A retrospective, single-center study from Israel assessed whether ibuprofen use in individuals with COVID-19 (*n* = 403) was associated with more severe disease, compared with use of paracetamol or no antipyretics^47^. Among the entire cohort, 44 patients (11%) needed respiratory support and 12 (3%) died; there was no significant difference in either outcome between the ibuprofen and non-ibuprofen groups, nor between ibuprofen users and exclusive paracetamol users. Kragholm et al. conducted a nationwide register-based study of patients in Denmark with COVID-19 to assess if prescription of ibuprofen affected the risk of severe disease^48^. The outcome assessed was a 30-day composite of severe COVID-19 diagnosis with acute respiratory syndrome, ICU admission, or death. The study found that there was no significant (*p* = 0.74) association between ibuprofen prescription claims and severe COVID-19; standardized absolute risks of the composite outcome for ibuprofen-prescribed vs. non-ibuprofen patients were 16.3% (95% CI 12.1–20.6) vs. 17.0% (95% CI 16.0–18.1). Similarly, in the Saudi Arabian study of COVID-19 patients reported by Abu Esba et al., acute use of ibuprofen was not associated with worse COVID-19 outcomes compared to non-NSAID users^38^.

Castro et al. used electronic health records to identify commonly prescribed medications that may be associated with a lower risk of morbidity among patients with COVID-19^49^. Records of 12,818 individuals from five US hospitals were assessed. Ibuprofen was associated with favorable outcomes, with users less likely to require hospitalization; among hospitalized patients, use of ibuprofen was associated with a lower risk of ICU admission and mortality.

A study of South Korean patients hospitalized with early-stage COVID-19 (*n* = 293) reported an increased likelihood of ibuprofen use among individuals with COVID-19 disease progression compared with those exhibiting improvement/stabilization^50^. However, after adjustment for confounding variables via propensity score matching, the authors found that ibuprofen was not a risk factor for progression. However, a study of 158 hospitalized patients from a single center in Iran reported a significant (*p* < 0.001) relationship between history of ibuprofen consumption before COVID-19 and the severity of the disease, as well as the mortality rate of patients with COVID-19^51^.

### Aspirin

Several of the studies identified included data for aspirin, and these specific findings are summarized below. Studies assessing the anti-platelet effects of aspirin in patients with COVID-19 are beyond the focus of the current review.

The large study of smartphone application users from the US, UK, and Sweden also included analysis of aspirin users^31^. The risk of contracting COVID-19 in this group was not significantly different from that among individuals not taking any NSAIDs, either before or after adjustment for confounders (HR after adjustment: 1.03 [95% CI 0.83–1.28]). In a US study of COVID-19 patients admitted to hospital, Chow et al. showed that the use of aspirin was associated with a more favorable disease course^52^. After adjustment for confounding variables, the risk of ICU admission was reduced by 43% (HR 0.63 [95% CI 0.31–0.90]) in users of aspirin compared with non-users. Similar reductions in the risks of mechanical ventilation (HR 0.56 [95% CI 0.37–0.85]) and in-hospital mortality (HR 0.53 [95% CI 0.31–0.90]) were also observed.

## Discussion

NSAIDs are a commonly used class of medication. They are affordable, widely available OTC, and have a well-established benefit-risk profile. NSAIDs provide benefits in managing COVID-19 symptoms, such as fever, body aches, and headache. In addition, many patients with chronic or recurrent inflammatory conditions rely on NSAIDs to manage their everyday symptoms. Therefore, some of these drugs, including ibuprofen, are on the WHO’s list of essential medicines to meet the most important needs in a health system.

Concerns about the potential of NSAID treatment to negatively impact COVID-19 outcomes were raised early in 2020. Since then, robust clinical data have been published, providing the opportunity to define the benefit/risk ratio of NSAID use with an evidence-based approach. The studies identified here represent the best available evidence to inform healthcare professionals and the scientific community. Of the 24 relevant publications identified, the overwhelming majority are consistent in showing that NSAIDs neither increase the likelihood of SARS-CoV-2 infection nor worsen clinical outcomes in patients with COVID-19. In addition, a meta-analysis demonstrated no increase in risk of mortality among NSAID users vs. non-NSAID users^29^. Another, very recent, systematic review and meta-analysis published after the cut-off of our search found no excess risk of SARS-CoV-2 positivity in patients exposed to NSAIDs^15^. In addition, in SARS-CoV-2-positive patients, exposure to NSAIDs was not associated with excess risk of hospital admission, death, or severe outcomes.

In contrast to the majority of investigations, four studies reported an association between NSAID use and worse COVID-19 outcomes. In the South Korean study of NSAIDs in hospitalized COVID-19 patients, there were differences in the baseline characteristics of NSAID users and non-users^46^. NSAID users were older, had more comorbidities, and used more comedications. Although Inverse Probability of Treatment Weighting was used to adjust for such differences, the number of patients exposed to NSAIDs (*n* = 285) might not have been sufficient to control for multiple confounders, and imbalances in unmeasured parameters such as smoking history or body mass index could have affected the results. Jehi et al. reported an increased risk of hospitalization among NSAID users, although the authors cautioned that their retrospective data did not enable robust conclusions to be drawn and highlighted uncertainty as to whether the observed relationships could have been caused by NSAIDs or underlying comorbidities^37^. While Samimagham et al. reported a significant relationship between history of ibuprofen consumption before COVID-19 and worse outcomes, the authors commented on the small sample size, focus on hospitalized patients, and lack of information on NSAID dose and indication for use/underlying conditions^51^. Reese et al. had the benefit of access to electronic health records data from >250,000 patients^42^. However, this report has not yet been peer reviewed and the authors acknowledge the possibility of incomplete capture of medications taken by the study participants. An update to this article, published in December 2021, accessed >850,000 health records and found no evidence of an association between NSAID use and risk of increasing COVID-19 severity^53^. Also, detection of significantly worsened disease among users of paracetamol was inconsistent with prior paracetamol studies and inconsistent with the suggestion that paracetamol should be used in preference to NSAIDs such as ibuprofen. While most studies have indicated the use of NSAIDs does not worsen outcomes for COVID-19 patients, the inherent limitations of these observational studies (including the fact that some of them are yet to be peer reviewed) require appropriate consideration when evaluating their results.

A number of review articles and editorials have been published on the possible effects of NSAIDs on the risk or severity of COVID-19. A predominant theme of these publications is the lack of evidence to suggest a mechanism by which NSAIDs (particularly ibuprofen) can worsen outcomes of COVID-19 infection. In fact, the hypothesis involving upregulation of ACE-2 appears to be contradicted by new evidence that NSAIDs do not affect expression of this enzyme^28^. The importance of basing therapeutic recommendations on robust evidence rather than speculative hypotheses has been emphasized^15^. The most recent editorial we identified, published in May 2021, concluded “NSAID use with COVID-19 appears to confer no increased risk of poorer outcomes”^54^.

To further increase complexity, recent data show genetic variations of cytochrome P450 liver oxidative enzyme systems can influence the metabolism of NSAIDs, potentially affecting their elimination from the body, their tissue concentrations, and the greater risks of adverse events^19^. Genetic variations differ between ethnic groups, meaning, for example, that the risk of higher-than-expected plasma drug levels may be greater in some populations than others. Future implementation of pharmacogenomics into clinical practice could, therefore, help optimize the use of NSAIDs in all patients including those with COVID-19.

Social media is a major influence in healthcare today, including the use of NSAIDs in COVID-19. There is a reasonable possibility of patients’ opinions being affected by information that is not scientifically founded^55,56^. At every opportunity, the importance of basing treatment decisions on consultation with healthcare professionals rather than unfounded information on social media should be emphasized.

Since the start of the pandemic, major health authorities across the world have published updated guidelines on clinical management of COVID-19 in an effort to combat misinformation. They consistently support the use of NSAIDs for treatment of COVID-19 symptoms, on the basis that there is no evidence of these drugs contributing to worsened outcomes. The WHO published its latest guidance in January 2021^12^. This document recommends that patients with mild COVID-19 “be given symptomatic treatment such as antipyretics for fever and pain, adequate nutrition and appropriate rehydration”, and states “at present, there is no evidence to indicate that there are severe adverse events in patients with COVID-19 as a result of NSAIDs”. On its website, the US Centers for Disease Control and Prevention (CDC) provides information specifically on COVID-19. In the section for healthcare professionals, the question “Do NSAIDs worsen the course of disease for people with COVID-19?” is addressed. In June 2021, the text stated “CDC is currently not aware of any evidence establishing a link between NSAIDs and worsening of COVID-19” and that they were continuing to monitor the situation^57^. On March 19, 2020, the US Food and Drug Administration (FDA) wrote they were “not aware of any scientific evidence connecting the use of NSAIDs with worsening of COVID-19 symptoms”^58^. They also reminded readers of the following warning: “the pharmacological activity of NSAIDs in reducing inflammation, and possibly fever, may diminish the utility of diagnostic signs in detecting infections”.

Advice from the EMA, UK National Institute for Health and Care Excellence, and the Australian Therapeutic Goods Administration is similar to that from the FDA, in that it has not been updated since March–May 2020. All these organizations stated that there was no scientific evidence supporting a link between NSAIDs and a worsening of COVID-19 and advocated continued use of NSAIDs as per the approved product information^59–61^. The Singapore Ministry of Health has also conducted a review and concluded that “there is no evidence suggesting additional harms with ibuprofen compared to paracetamol when used to manage symptoms of upper respiratory tract infections”^62^. Provisional recommendations from the European Alliance of Associations for Rheumatology on the management of rheumatic disease during the pandemic, dated May 2020, stated that NSAID treatment should be continued in patients without COVID-19^63^. This group also wrote that, based on current knowledge, NSAIDs could be used during the pandemic without additional risk. Similar guidance was published by the American College of Rheumatology (ACR) in December 2020, recommending that NSAID treatment should be continued in patients with or without exposure to SARS-CoV-2^64^. The ACR also stated that possible associations between NSAID use and worse COVID-19 outcomes are yet to be substantiated.

There are several potential limitations to this literature review. This was a narrative literature review, and systematic principles were therefore not followed. As part of the review, the risk of bias in the included studies was not assessed. In addition, the published studies vary considerably in several ways, including geographical setting, medical setting (hospitalized patients, outpatients, primary care), number of patients included, medications and doses (e.g., prescription and OTC doses), outcomes assessed, and statistical methods applied. However, these differences add strength by ensuring the data provide insight from multiple different perspectives. In particular, studies have been identified from multiple regions of the world, providing a global aspect of the challenge and, importantly, showing the same responses are reported regardless of ethnicity.

In summary, from observational studies conducted to date, there is no evidence of an association between the use of NSAIDs and increased susceptibility to COVID-19 or increased disease severity, and no plausible causative mechanism by which NSAIDs would increase susceptibility to or the severity of COVID-19 illness. This is reflected in the current recommendations from major public health authorities across the world, which support NSAID use for analgesic or antipyretic treatment during COVID-19. Although further data (e.g., from randomized controlled trials) are required to fully elucidate the relationship between NSAID therapy and COVID-19 severity, there is no basis on which to restrict or prohibit use of these drugs by consumers or patients to manage their health conditions and symptoms during the pandemic. As NSAID users often have clinical characteristics associated with worsened outcomes in COVID-19, restricting access or prohibiting use of these drugs during the pandemic may have a negative impact on patients who consequently are likely to use opioids or subtherapeutic treatment options.

## Data Availability

Data included in the review is all sourced from published information and publicly available.
